# Immune response in piglets orally immunized with recombinant *Bacillus subtilis* expressing the capsid protein of porcine circovirus type 2

**DOI:** 10.1186/s12964-020-0514-4

**Published:** 2020-02-11

**Authors:** Shuai Zhang, Chunxiao Mou, Yanan Cao, En Zhang, Qian Yang

**Affiliations:** MOE Joint International Research Laboratory of Animal Health and Food Safety, College of Veterinary Medicine, Nanjing Agricultural University, Wei gang 1, Nanjing, Jiangsu 210095 People’s Republic of China

**Keywords:** *Bacillus subtilis* WB800N, Porcine circovirus type 2 (PCV2), Capsid protein, Dendritic cells, Neonatal piglets, Immune responses

## Abstract

**Background:**

Porcine circovirus type 2 (PCV2) is the causative agent of postweaning multisystemic wasting syndrome, and is associated with a number of other diseases. PCV2 is widely distributed in most developed swine industries, and is a severe economic burden. With an eye to developing an effective, safe, and convenient vaccine against PCV2-associated diseases, we have constructed a recombinant *Bacillus subtilis* strain (*B. subtilis*-Cap) that expresses the PCV2 capsid protein (Cap).

**Methods:**

Electroporation of a plasmid shuttle vector encoding the PCV2 Cap sequence was use to transform *Bacillus subtilis*. Flow cytometry was used to evaluate in vitro bone marrow derived dendritic cell (BM-DC) maturation and T cell proliferation induced by *B. subtilis*-Cap. Orally inoculated piglets were used for in vivo experiments; ELISA and western blotting were used to evaluate *B. subtilis*-Cap induced PCV2-specific IgA and IgG levels, as well as the secretion of cytokines and the expression of Toll-like receptor 2 (TLR2) and Toll-like receptor 9 (TLR9).

**Results:**

We evaluated the immune response to *B. subtilis*-Cap in vitro using mouse BM-DCs and in vivo using neonatal piglets orally inoculated with *B. subtilis*-Cap. Our results showed that the recombinant *B. subtilis*-Cap activated BM-DCs, significantly increased co-stimulatory molecules (CD40 and CD80) and major histocompatibility complex II, and induced allogenic T cells proliferation. Piglets immunized with *B. subtilis*-Cap had elevated levels of PCV2-specific IgA in the mucosal tissues of the digestive and respiratory tract, and PCV2-specific IgG in serum (*P* < 0.05 or *P* < 0.01). Ileal immunocompetent cells, such as the IgA-secreting cells (*P* < 0.01), intestinal intraepithelial lymphocytes (IELs) (*P* < 0.01), CD3^+^ T lymphocytes (*P* < 0.01) and CD4^+^ T lymphocytes (*P* < 0.01) increased significantly in the *B. subtilis*-Cap immunized piglets. Additionally, *B. subtilis*-Cap inoculation resulted in increased the expression of TLR2 and TLR9 (*P* < 0.01), and induced the secretion of cytokines IL-1β, IL-6, interferon-γ, and β-defensin 2 (*P* < 0.01).

**Conclusions:**

We constructed a prototype PCV2 vaccine that can be administered orally and elicits a more robust humoral and cellular immunity than inactivated PCV2. *B. subtilis*-Cap is a promising vaccine candidate that is safe, convenient, and inexpensive. Further in vivo research is needed to determine its full range of efficacy in pigs.

**Graphical abstract:**

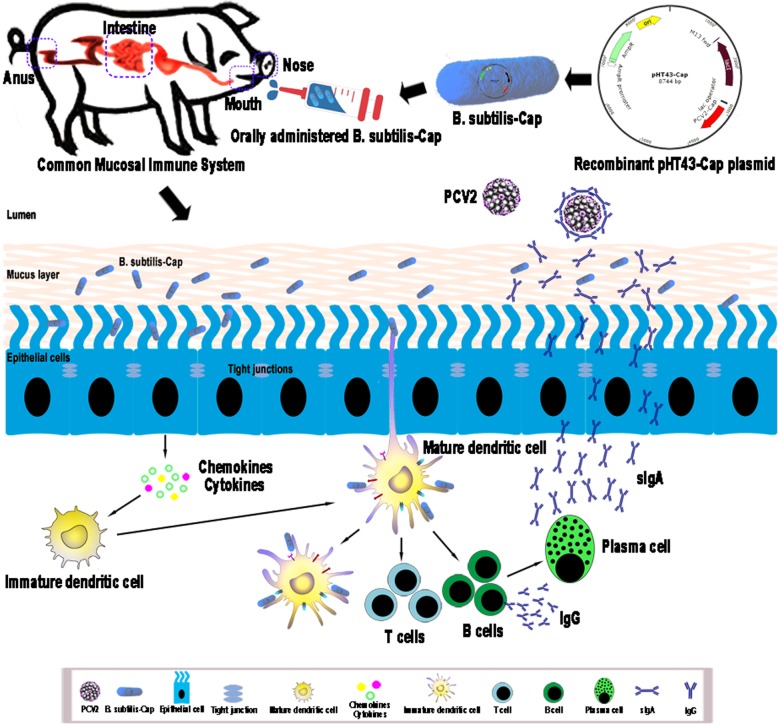

## Background

Porcine circovirus (PCV) is a member of the genus *Circovirus* in the family *Circoviridae* [1]. Currently three genotypes of PCV are recognized: types 1, 2, and 3. PCV2 is a small (about 17 nm), icosahedral, non-enveloped virus containing a single-stranded negative sense DNA genome [2]. The DNA is approximately 1700 nucleotides and includes two open reading frames (ORFs) [3–5]. ORF1 encodes two replication-associated proteins (Rep and Rep’) and ORF 2 encodes the viral capsid protein (Cap). Cap is the principal immunogenic protein available for the induction of PCV-neutralizing antibodies [6–8]. PCV2 is the causative agent of postweaning multisystemic wasting syndrome (PMWS) [9–11]. It is widely distributed in most of the world’s swine populations and may have an association with other porcine circovirus associated diseases (PCVAD) [12], such as porcine respiratory disease complex (PRDC), porcine dermatitis and nephropathy syndrome (PDNS), reproductive failure, and congenital tremors [13–16]. PCV2 preferentially resides in immune cells, such as macrophages and dendritic cells, and impairs their function. PCV2 infection usually accompanies lymphocyte or monocyte depletion which causes further immune suppression [17, 18]. Particularly in piglets, the immunosuppression caused by PCV2 often results in concurrent infection with other pathogens, such as porcine reproductive respiratory syndrome virus (PRRSV), porcine parvovirus (PPV), *Haemophilus parasuis*, or *Mycoplasma hyopneumoniae*. Together, these PCV-associated diseases impose an enormous economic burden on swine industries across the world [19, 20].

PCV2 infection may initially occur on mucosal surfaces, especially the respiratory or intestinal tract [21–24]. However, the vaccines now available cannot effectively induce mucosal immunity against PCV2 infection when administered parenterally. Commercial PCV2 vaccines (delivered intramuscularly) that include inactivated whole PCV2 virus, a subunit of open reading frame 2, or inactivated chimeric PCV1–2, can reduce clinical signs and increase production parameters in farms with PMWS [25–28]. However, these vaccines do not prevent the infection or transmission of PCV2 completely [25]. In addition, intramuscular injection involves laborious and time-consuming procedures, causes inflammatory responses, and risks management-derived stress reactions that hamper animal growth and degrade meat quality [29]. There is an urgent need for a safe and efficient PCV2 vaccine that is both convenient to administer and inexpensive. Compared with traditional vaccines, mucosal vaccines are administered orally or intranasally, cause less stress, and stimulate effective mucosal immune responses [30–32]. It would therefore be highly desirable to develop a PCV2 vaccine that is delivered without injection and is able to induce mucosal immune responses.

*Bacillus subtilis* (*B. subtilis*) is a non-pathogenic gram-positive bacterium that has been used as an additive to improve the growth performance and the feed conversion ratio in animals [33–35]. It has also been widely used as a probiotic bacterium to induce non-specific immune responses against infection, such as increasing IgA production and regulating the balance of the Th1 and Th2 pathways [36, 37]. Lysozyme, phospholipase A and other enzymes found in the saliva along with intestinal proteases, pepsin, and the highly acidic gastric environment (pH 1–2) present a particularly harsh environment for orally administered vaccines [38], such as the inactivated PCV2. The ability of *B. subtilis* to survive under extreme conditions, such as high temperatures, desiccation, and exposure to noxious chemicals [39], and *B. subtilis* is highly stable and ismable to withstand the chemical and enzymatic conditions of the intestinal tract, which makes it attractive as a therapeutic molecule and oral vaccine vehicle for delivery of immunogens to intestinal mucosa [40, 41].

In this study, we used *B. subtilis* WB800N to construct a recombinant bacterial strain that expressed the Cap protein of PCV2 virus. To determine if the recombinant can be used as an effective oral mucosal vaccine, we administered it orally to piglets and then tested their local mucosal and systemic immune responses.

## Methods

### Bacteria, plasmids, virus, and animals

*B. subtilis* WB800N and the pHT43 expression vector [42] (*E. coli-B. subtilis* shuttle vector) used to express recombinant proteins in *B. subtilis* were kindly provided by Dr. Xuewen Gao. The pMD-19 T-Cap cloning vector was previously constructed in our laboratory.

Porcine circovirus 2 strain DBN-SX07–2 (GenBank: HM641752.1) was kindly provided by Dr. Ping Jiang, propagated on porcine kidney (PK) 15 cells, and stored at − 70 °C. Virus cultures were filtered through a 0.22 μm filter, and concentrated by ultra-centrifugation at 10,000×g for 2.5 h, then purified on a discontinuous sucrose density gradient at 10,000×g for 2.5 h. The purified PCV2 was inactivated by exposure to ultraviolet light for 4 h then tested for complete loss of infectivity by inoculation onto PK15 cells which were subsequently passaged 3 times. Purified virus concentrations were measured by BCA protein assay.

4-week-old male C57BL/6 mice were obtained from the Animal Research Center of Yangzhou University (Yangzhou, China). Twenty-four Duroc Landrace Yorkshire piglets, obtained from healthy primiparous sows free from TGEV, PEDV, PCV2, and PRRSV, were isolated from the sows at birth and fed artificial milk, containing no maternally derived antibodies. At 5 days of age they were divided into four groups and maintained in separate rooms. The animal studies were approved by the Institutional Animal Care and Use Committee of Nanjing Agricultural University (Nanjing, China) and followed the National Institutes of Health’s guidelines for the performance of animal experiments.

### Reagents and antibodies

Plasmid isolation and gel elution kits were purchased from Axygen Biosciences (Union City, CA, USA). DNA size markers were purchased from Novoprotein (DM028-01A). DNA polymerase and Taq polymerase were purchased from TaKaRa Biotechnology Corporation (Dalian, Japan). Restriction enzymes were purchased from New England Biolabs (England). ClonExpress II one step cloning kit (Vazyme). DNA used in the experiments was quantified using a NanoDrop 2000 spectrophotometer (Thermo Scientific USA). The antibiotics and the concentrations used in culturing *B. subtilis* were kanamycin (50 μg/ml), chloromycetin (15 μg/ml), ampicillin (100 μg/ml), isopropy-β-D-thiogalactopyranoside (IPTG 0.1 mM). RPMI 1640 medium, penicillin and streptomycin were purchased from Invitrogen (Grand Island, NY, USA). Fetal bovine serum (GIBCO), recombinant GM-CSF (granulocyte-macrophage colony-stimulating factor), and IL-4 were purchased from PeproTech (Rocky Hill, NJ). LPS (from *E. coli* 026:B6) was obtained from Sigma-Aldrich (St Louis, MO, USA). Red blood cell lysing buffer (Beyotime, China). T cell isolation kit (Miltenyi Biotech, Germany). RIPA lysis buffer and SDS-PAGE sample loading buffer (5×) were obtained from FcMACS (Nanjing, China). The BCA protein assay kit was obtained from Thermo Scientific (USA).

Fluorescent-labeled anti-mouse mAbs CD40-FITC, CD80-FITC, and MHC II-FITC were used for flow cytometry and were purchased from eBioscience (San Diego, CA, USA). Mouse anti-Cap mAbs were kindly provided by Dr. Ping Jiang [43]. Dylight 488 goat anti-mouse IgG (H + L) (MultiSciences (Lianke) Biotech, CO., LTD). The florescent dye carboxyfluorescein succinimidyl ester (CFSE) was obtained from Invitrogen (Grand Island, NY, USA). Rabbit anti-pig CD3 (SP7) mAbs was purchased from Abcam (Hongkong). FITC rabbit anti-pig CD4a was purchased from Santa Cruz Biotech. Mouse anti-pig CD8 alpha antibody (76–2-11) was purchased from Novus. SABC-POD (rabbit and mouse IgG) kits and peroxidase substrate kits were purchased from BOSTER (Wuhan, China). Rabbit anti-human TLR2 and TLR9 polyclonal antibodies were purchased from Thermo Scientific USA. Rabbit anti-GAPDH and goat anti-rabbit IgG-HRP were from BioWorld Technology, Inc. (St. Louis Park, MN, USA). Enhanced chemiluminescence reagents were purchased from New Cell & Molecular Biotech Co., Ltd. (China). Mouse PCV2 IgA and IgG ELISA kits, porcine PCV2 IgA and IgG ELISA kits, the porcine interleukin 6 (IL-6) ELISA kit, the porcine interleukin 10 (IL-10) ELISA kit, and the porcine interferon-γ (IFN-γ) ELISA kit were purchased from SenBeiJia Biological Technology Co., Ltd. (Nanjing, China). The pig IL-1 beta ELISA kit (ab 100,754) and the pig TNF alpha ELISA kit (ab 100,756) were acquired from Abcam (Hongkong). The porcine defensin beta 2 (DEFb2) ELISA kit was purchased from Bio-Swamp.

### Construction of pHT43-cap and recombinant *B. subtilis*-cap strains

**Fig. 1:**
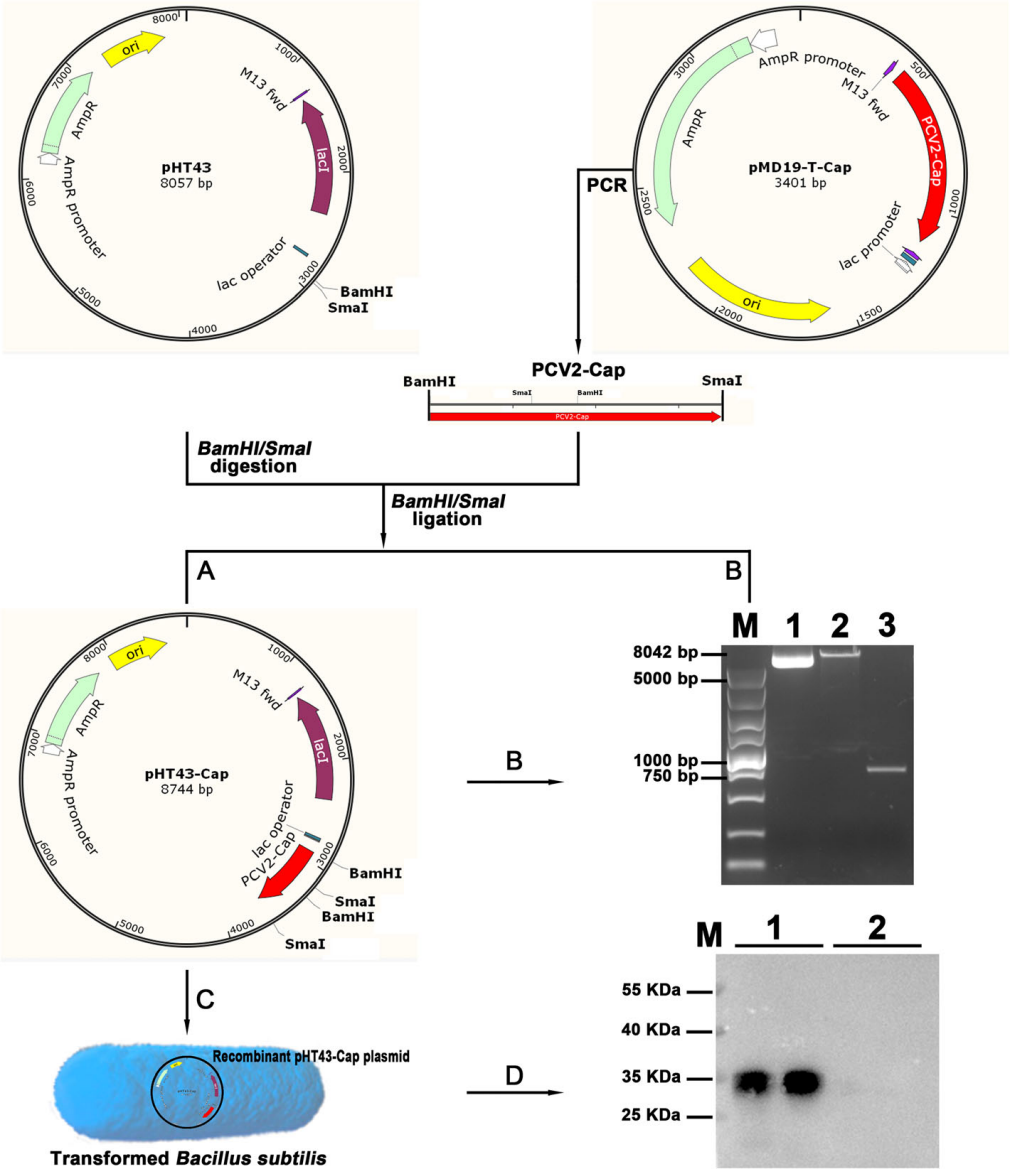
Schematic of pHT43-Cap construction and protein expression. **a** The Cap sequence was amplified from pMD19-T-Cap, and inserted into pHT43 between the BamHI and SmaI sites, generating plasmid pHT43-Cap. **b** Agarose gel of pHT43-Cap, pHT43, and Cap fragment amplified from pMD19-T-Cap in lanes 1, 2, and 3 respectively. Plasmids were digested with BamHI and SmaI before electrophoresis. Lane M is a 5000 bp DNA ladder. **c** pHT43 (vector control) and pHT43-Cap recombinant plasmids were transformed into *B. subtilis* by electroporation. **d** Western blotting analysis of the PCV2 Cap protein expressed in *B. subtilis*-Cap, lanes 1. Extracts of *B. subtilis* (transfected with pHT43) were run in lanes 2, and lane M is protein molecular weight ladder

To obtain a recombinant strain of *B. subtilis* WB800N with the ability to express PCV2 Cap protein, the plasmid shuttle vector pHT43-Cap was constructed (Fig. 1). First, the PCV2 Cap sequence (702 bp, GenBank accession number ADM16685.1) was amplified from pMD-19 T-Cap using the primers F:5′-tcagccgtaggatccatgacgtatccaagg-3′ (BamHI site is underlined) and R:5′-ggcgggctgccccgggttagggtttaagtg-3′ (SmaI site is underlined). The *E. coli-B. subtilis* shuttle vector pHT43 was prepared for use by digestion with BamHI and SmaI. The Cap amplicon was purified, digested with BamHI and SmaI, then inserted downstream of the Pgrac promoter region in pHT43 using a ClonExpress II one step cloning kit to generate the recombinant pHT43-Cap shuttle vector in *E. coli* DH5a cells (Fig. 1a). The structure of pHT43-Cap was confirmed by DNA sequencing (Sangon Biotech).

The pHT43 vector (control) and the pHT43-Cap recombinant plasmid were transformed into *B. subtilis* by electroporation as previously described [42, 44]. The transformed strains were designated *B. subtilis* and *B. subtilis*-Cap, respectively. Briefly, 50 ng of recombinant pHT43-Cap plasmid was gently mixed with 60 μL of competent *B. subtilis* for 1 min at 4 °C. The mixture was transferred into a pre-cooled electroporation cuvette (inter-electrode distance of 0.1 cm) (Bio-Rad, Hercules, CA, USA) and subjected to a single electric pulse (22 KV/cm, 25 μF, 200 Ω). The cells were then cultured overnight at 37 °C, and positive colonies were selected based on growth on 15 μg/mL chloramphenicol antibiotic agar Luria–Bertani (LB) plates (10 g/L tryptone, 5 g/L yeast extract, 10 g/L NaCl, and 20 g/L agar).

### Expression of cap protein by the recombinant *B. subtilis*

To detect expression of the Cap protein, a single transformed colony was inoculated into LB medium containing chloramphenicol (15 μg/mL) and incubated overnight at 37 °C with agitation at 180 rpm. Fresh LB medium (100 ml) was inoculated with the overnight culture, to an initial cell density (OD_600_) of 0.1. Pgrac in pHT43 is derived from a fusion between the strong promoter that precedes the groESL operon of *Bacillus subtilis* and the lac operator, enabling the IPTG-inducible expression of downstream genes. When the culture reached log-phase (OD_600_ 0.7–0.8), IPTG was added to 0.1 mM [45] and then incubated for 8 h. The bacteria were collected by centrifugation at 3000×g for 10 min at 4 °C, and cells were disrupted by sonication. For immunodetection of the heterologous Cap protein, western blots were prepared and incubated with mouse anti-Cap specific antibody (1:1000), followed by horseradish peroxidase (HRP)-conjugated rabbit anti-mouse IgG (1:5000). Binding was detected using enhanced chemiluminescence.

### Preparation of recombinant *B. subtilis* for immunization

The recombinant *B. subtilis*-Cap and corresponding empty plasmid control (*B. subtilis-*pHT43) were cultured in LB medium or on LB plates fortified with 1.5% agar and appropriate antibiotic at 37 °C. Bacterial cultures were centrifuged at 3000×*g* for 10 min at 4 °C and washed three times with sterile phosphate-buffered saline (PBS). For use in oral immunization, the washed pellets were resuspended to 1 × 10^10^ CFU/ml in sterile PBS supplemented with 1% sucrose. Bacterial titers (colony forming units or CFU) were determined by plate counts. Successive dilutions (the values of CFU between 30 and 300) of each strain were selected for calculation.

### Isolation and culture of mouse bone marrow derived dendritic cells

Bone marrow derived dendritic cells (BM-DCs) were generated as previously reported [46]. Briefly, bone marrow cells were flushed from the tibias and femurs of 4-week old C57BL/6 mice, treated with red blood cell lysing buffer and cultured in RPMI 1640 medium supplemented with 10% heat-inactivated FBS, 1% streptomycin and penicillin, 10 ng/ml GM-CSF and IL-4) in 6-well plates (Corning, Cambridge, MA) and incubated at 37 °C. After 3 days, non-adherent granulocytes were removed when the spent medium was replaced with fresh. On day 6, non-adherent and loosely adherent DC aggregates were harvested and subcultured overnight. The non-adherent cells, >90%, were used as immature DCs for studies on day 7, see below.

### Exposure of DCs to the recombinant *B. subtilis*-cap

On day 7, immature DCs were seeded into 24-well plates (5 × 10^5^ cells/well) and treated with 10 ng/ml of LPS (positive control) or recombinant *B. subtilis* (10^7^ CFU/well) for 24 h at 37 °C. Cells were then washed twice with cold PBS and stained with fluorescent mAbs specific for mouse CD40, CD80, and MHC II for 0.5 h at 4 °C as per manufacturer’s guidelines. Cells were washed three times with PBS then subjected to flow cytometry.

### Allogenic mixed lymphocyte reaction

The functional activity of DCs was reflected in the primary allogeneic mixed lymphocyte reaction assay. T cells were purified from the mesenteric lymph nodes of allogeneic BALB/c mice by using a T cell isolation kit and then labeled with CFSE according to manufacturer’s instructions. Responder T cells (5 × 10^5^/well) were co-cultured with BM-DCs (DCs/T cell ratios of 1:1) in 24-wells plates for 5 days in 5% CO_2_ incubator at 37 °C and then subjected to flow cytometry analysis.

### Immunization schedule and sample collection

To evaluate the immunogenicity of recombinant *B. subtilis*-Cap strain as an oral vaccine, 24 neonatal Yorkshire piglets were randomly allocated to four immunization groups: Ctrl, *B. subtilis*, inactivated PCV2, and *B. subtilis*-Cap; all immunizations were done orally. Each piglet in the *B. subtilis*-treated groups was administered *B. subtilis* preparation (10^10^ CFU/ml, 10^10^ CFU/kg *B. subtilis*) 1 ml on day 0, 2 ml on day 7 and 8 ml on day 35, while the piglets in the Ctrl group were given the same volumes of PBS, the piglets in the inactivated PCV2 group were given 100 μg/dose of inactivated PCV2. Serum, oropharyngeal swabs, nasal swabs, and feces were collected at weekly intervals after the second immunization, and piglets were not allowed to ingest anything for the 2 h previous to sample collection. Serum was collected from the jugular vein of the piglets. Oropharyngeal samples were taken by allowing the piglets to bite a swab four times. Nasal swabs were introduced 3 cm into the nose. 0.3 g of feces were collected using a cotton swab introduced 4 cm into the rectum. Samples were transported on dry ice to the research lab, where they were placed in tubes containing 0.5 ml cold PBS, vortexed at least 30 s and clarified by centrifugation at 2500×g for 10 min at 4 °C. The clarified washes were stored at − 70 °C until use for PCV2-specific IgA and IgG detection by ELISA, see below. All piglets were sacrificed on day 39, ileums were removed and transported on dry ice to the research lab. 100 mg of frozen ileal tissue from each piglet was homogenized in 1 ml of RIPA buffer (containing phosphatase and protease inhibitors) for western blot or 1 ml of PBS for cytokine detection with a Brinkman homogenizer at low speed for 2–3 min. Tissue RIPA buffer homogenates were then centrifuged at 12,000×g for 10 min at 4 °C and tissue PBS homogenates were then centrifuged at 2500×g for 10 min at 4 °C. Supernatants were collected and stored at − 70 °C until further use. The protein concentration of the supernatants was determined by BCA protein assay according to the manufacturer’s directions.

### Serum neutralization assays

PCV2 neutralizing activity in sera from swine was detected on day 21 and day 28 by a fluorescent focus neutralizing assay as previously described [8]. Briefly, the serum samples were heat inactivated at 56 °C for 30 min and fifty microlitres of the serum samples were serially diluted in DMEM medium (pH 7.0) in two-fold with a starting dilution of 1:2 to 1:4096. Then 200 TCID_50_ of PCV2 (4 × 10^3^ TCID_50_ ml^− 1^, 50 μl) were mixed with equal volume of serum. After incubation for 1 h at 37 °C, the mixtures were added to a 96-well plate containing 40–50% confluent PK-15 cells and incubated at 37 °C for 1 h. Then, the inoculum were removed, cells were washed with DMEM three times and complete medium were added for incubation. After 72 h, the cells were washed twice with PBS and fixed with cold acetone/methanol (1/1 v/v) at − 20 °C for 20 min, and blocked with 3% BSA in PBS for 1 h at room temperature, and then air-dried. The cells were incubated with anti-Cap monoclonal antibodies diluted at 1:100 with PBS containing 1% BSA for 1 h at 37 °C, and then followed by staining with Dylight 488 goat anti-mouse IgG (H + L) (1:200) as second antibody at 37 °C for 1 h in the dark. All cells were washed 3 times with PBS and read under a fluorescent microscope. Cells with Dylight 488 staining were recorded as infected. Once a plate was validated, neutralizing antibody (NA) titers were determined as the reciprocal of the highest serum dilution at 50% or greater fluorescent focus reduction in the infected cell cultures under a fluorescent microscope.

### Levels of PCV2-specific IgA, IgG, and cytokines in piglet samples

Levels PCV2-specific IgA from saliva, the nasal cavity, and feces and IgG in serum were measured by ELISA according the manufacturer’s recommended protocol. The signal to noise (S/N) value was calculated as follows:

$$ \mathrm{S}/\mathrm{N}=\frac{\left(\mathrm{OD}450\ \mathrm{of}\ \mathrm{sample}\ \mathrm{well}-\mathrm{OD}450\ \mathrm{of}\ \mathrm{bank}\ \mathrm{control}\ \mathrm{well}\right)}{\left(\mathrm{OD}450\ \mathrm{of}\ \mathrm{negative}\ \mathrm{control}\ \mathrm{well}-\mathrm{OD}450\ \mathrm{of}\ \mathrm{bank}\ \mathrm{control}\ \mathrm{well}\right)} $$

Cytokine levels in the ileum suspensions were measured using commercial ELISA kits according to the manufacturer’s instructions. Absorbance was measured at 450 nm with an automated ELISA reader.

### Histological examination of intestinal intraepithelial lymphocytes (IELs)

Ileal tissues from slaughtered piglets were fixed with Bonn’s liquid, embedded in paraffin, and serially sectioned (5 μm per section). Five non-successive sections from the same location of ileum tissue were selected and stained with hematoxylin–eosin (H/E). Tissue sections were visualized with a 40× objective on an Olympus BH-2 microscope. Measurement of the IELs was done as described [47]. Briefly, ten well-oriented villi and crypts, the longest in each field, were selected and the number of IELs counted; the means of these counts were calculated to yield a single value per pig. All the data were used for statistical analysis and expressed as the average number of cells.

### Immunohistochemical detection of IgA secreting cells, CD3^+^, CD4^+^, and CD8^+^ T lymphocytes

Paraffin sections were dewaxed in xylene and rehydrated in decreasing concentrations of ethanol. For immunohistochemical staining, antigen retrieval was performed for 30 min with citrate buffer at pH 6.0 in a Decloaking Chamber at 95 °C. Slides were blocked with 5% normal goat serum then incubated with primary antibody (1:100) overnight at 4 °C in a humidified chamber. Sections incubated in buffer only served as the negative control. The SABC-POD (rabbit or mouse IgG) kit and peroxidase substrate kit were used for amplification and visualization of signal, respectively. Following each incubation step, sections were washed 4 times, each wash was in fresh PBS-Tween. The sections were visualized with an Olympus BH-2 microscope, and ten fields were selected from each section (40×). The regions that contained IgA secreting cells, CD3^+^, CD4^+^, and CD8^+^ T lymphocytes were counted using the Image-Pro Plus analysis program (Cambridge, UK), data are expressed as the average IOD of cells.

### Extraction of total protein and western blotting assay

TLR2 and TLR9 protein levels in ileal tissue homogenates were determined by western blot. Equal amounts of protein were mixed with 5× SDS-PAGE sample loading buffer, incubated 10 min in a boiling water bath then separated on a 10% SDS-PAGE gel. Proteins were transferred onto a 0.22 μm-pore polyvinylidene difluoride membrane (PVDF; Merck Millipore) at 110 V for 1 h using a wet-transfer apparatus in an ice bath. The membranes were blocked for 2 h with 5% skim milk in PBST (PBS-0.1% Tween-20) and then incubated with primary antibodies (1:1000) at 4 °C overnight. After washing with PBST/0.1% BSA, the membranes were incubated with horseradish peroxidase (HRP)-conjugated secondary antibodies (1:5000) for 1 h at room temperature. After additional washing, bound conjugates were detected by enhanced chemiluminescence. Proteins were visualized using an electro-chemiluminescence visualization system (Tanon, Shanghai, China) and then the net intensities of the individual bands were measured using Quantity One (Quantity One 1-D Analysis Software 170–9600, Bio-Rad). The density of the bands was measured and GAPDH was used as the loading control, and TLR2 and TLR9 protein expression were normalized to GAPDH.

### Statistical analysis

Data are presented as means ± standard deviation (SD) from three independent experiments. Statistical analysis was performed using the Statistical Program for Social Sciences (SPSS) 16.0. Significant differences between control and experimental groups were analyzed by Student’s *t*-test and one-way ANOVA. Differences were considered statistically significant at * 0.01 < *p* < 0.05, ** *p* < 0.01.

## Results

### Construction of pHT43-cap shuttle plasmid and expression of cap protein in recombinant *B. subtilis*

The PCV2 Cap sequence was inserted downstream from the Pgrac site in pHT43, resulting in pHT43-Cap (Fig. 1a). pHT43-Cap and empty pHT43 were transformed into *B. subtilis* by electroporation. A western blot of extracts of transformed *B. subtilis*-Cap and *B. subtilis*-pHT43 (named *B. subtilis*) probed with mouse anti-Cap mAbs, revealed a 30 kDa band in *B. subtilis*-Cap and not in *B. subtilis* (Fig. 1d). 30 kDa corresponds to the molecular mass of the PCV2 Cap protein. These results demonstrate that the Cap protein was expressed in *B. subtilis* with effective antigenicity.

### *B. subtilis*-cap stimulation of BM-DC maturation in vitro

**Fig. 2:**
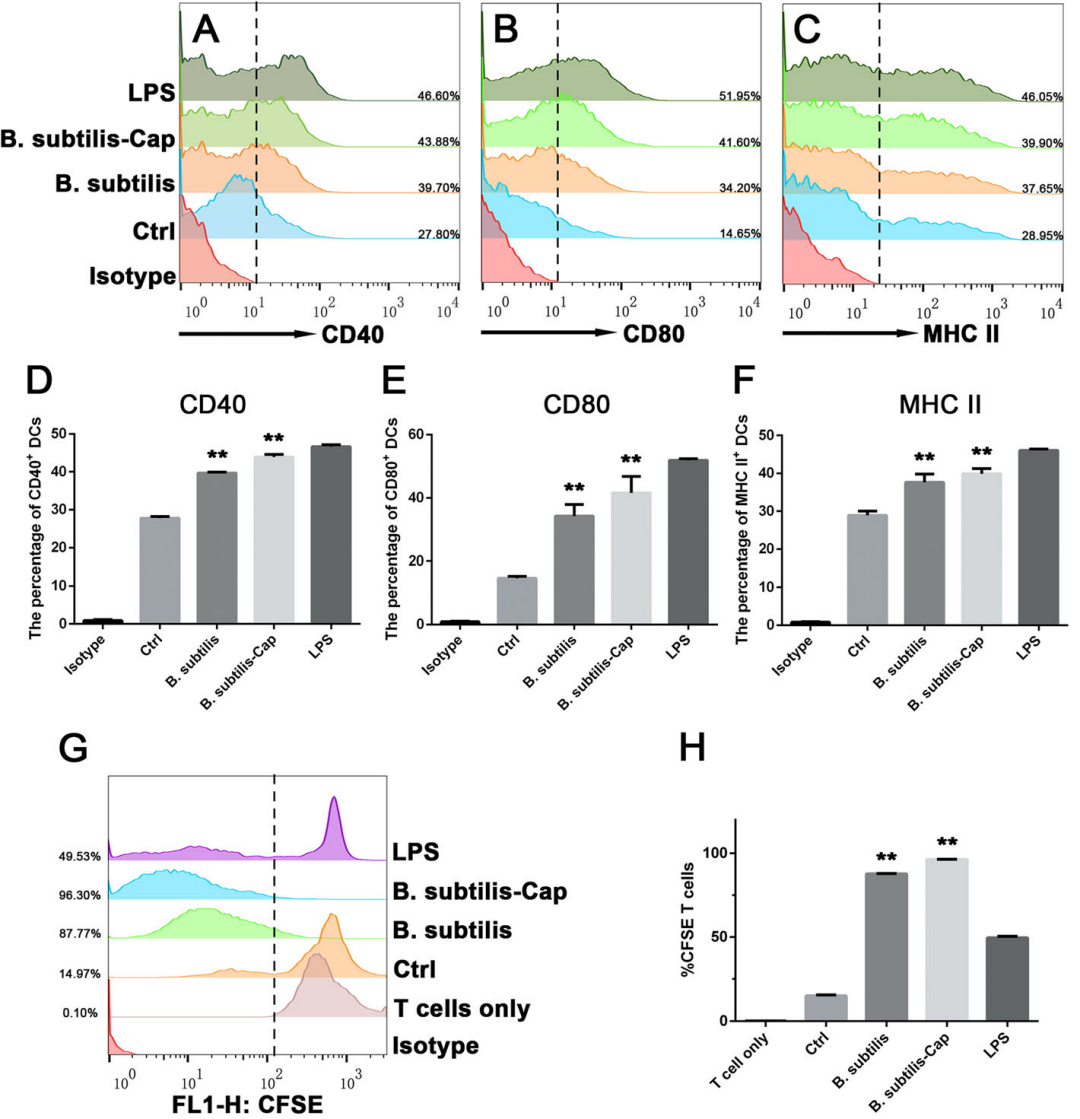
*B. subtilis*-Cap stimulates phenotypic alteration of BM-DCs and promotes T cells proliferation in vitro. **a**-**f***B. subtilis*, *B. subtilis*-Cap, and LPS, were incubated with mouse BM-DCs for 24 h. Expression of CD40, CD80, and MHCII were analyzed by flow cytometry. Untreated BM-DCs served as a negative control. **g** and **h** Mixed lymphocyte reaction (MLR) experiments. *B. subtilis*, *B. subtilis*-Cap, LPS, or PBS treated BM-DCs were co-cultured with carboxyfluorescein succinimidyl ester (CFSE)-labeled allogeneic T lymphocytes (DC:T cell ratio, 1:1) for another 5 days. Untreated BM-DCs co-cultured with T lymphocytes served as a negative control. T lymphocytes proliferation was evaluated by flow cytometry. Data are presented as means ± SD from three independent experiments. (* 0.01 < *p* < 0.05, ** *p* < 0.01)

DCs play an important role in capturing antigens and activating the immune response. When immature DCs contact an antigen they activate into mature cells and up-regulate the expression of co-stimulatory and antigen presentation associated factors [48]; this phenotypic maturation of DCs is essential for T cells activation. To determine whether *B. subtilis*-Cap could stimulate the maturation of bone marrow dendritic cells, we used specific antibodies to detect the surface expression of CD40, CD80, and MHCII in mouse BM-DCs by FACS. As shown in Fig. 2a-f, compared to immature BM-DCs, the expression of CD40, CD80, and MHC II were significantly increased (*P* < 0.01) after being stimulated by *B. subtilis*, *B. subtilis*-Cap or LPS. *B. subtilis*-Cap elicited a greater stimulatory response than *B. subtilis* or Ctrl.

The immune stimulatory effects of DCs on T cells and T lymphocyte proliferation are crucial to the adaptive immune response. After stimulation with *B. subtilis*, *B. subtilis*-Cap, and LPS in vitro, an allogenic mixed lymphocyte reaction (MLR) was performed to determine the ability of activated BM-DCs to induce allogenic T cell proliferation. As shown in Fig. 2g and h, BM-DCs stimulated by *B. subtilis* and *B. subtilis*-Cap increase allogeneic T cells proliferation compared with those in the untreated control group (*P* < 0.01). Previous studies have reported that the ratio of CD4^+^ T cells to CD8^+^ T cells plays a central role in the induction of efficient immune responses against diseases such as human immunodeficiency virus and some cancers [49–51]. The ratio of CD4^+^ to CD8^+^ T cells in the *B. subtilis*-Cap group was significantly higher than in other groups (Additional file 1: Figure S1), demonstrating that the recombinant *B. subtilis*-Cap effects an efficient immune response against PCV2.

### Immune response in piglets orally inoculated with *B. subtilis*-cap

We evaluated the mucosal and systemic immune responses in Balb/c mice orally inoculated with *B. subtilis*, *B. subtilis*-Cap, and inactivated PCV2. PCV2-specific IgA levels in ileum fluids from the *B. subtilis*-Cap, and inactivated PCV2 dosed mice, rose in concert over the course of 21 days, then declined. In mice inoculated with *B. subtilis*-Cap, the rise was steeper and the decline was shallower than in mice dosed with inactivated PCV2. On day 35 post inoculation the IgA levels in the intestines of *B. subtilis*-Cap dosed mice were still elevated, while in mice dosed with inactivated PCV2, the IgA levels had declined to background (Additional file 2: Figure S2a). PCV2-specific IgG levels in serum from the *B. subtilis*-Cap, and inactivated PCV2 dosed mice rose in concert over the course of 14 days, between day 14 and 28, the serum IgG levels in the *B. subtilis*-Cap dosed mice continued to rise, then slightly decline between day 28 and 35, while in the mice dosed with inactivated PCV2, serum IgG levels declined to background after day 14 (Additional file 2: Figure S2b). These results demonstrated that recombinant *B. subtilis*-Cap could effectively stimulate an immune response against PCV2.

**Fig. 3:**
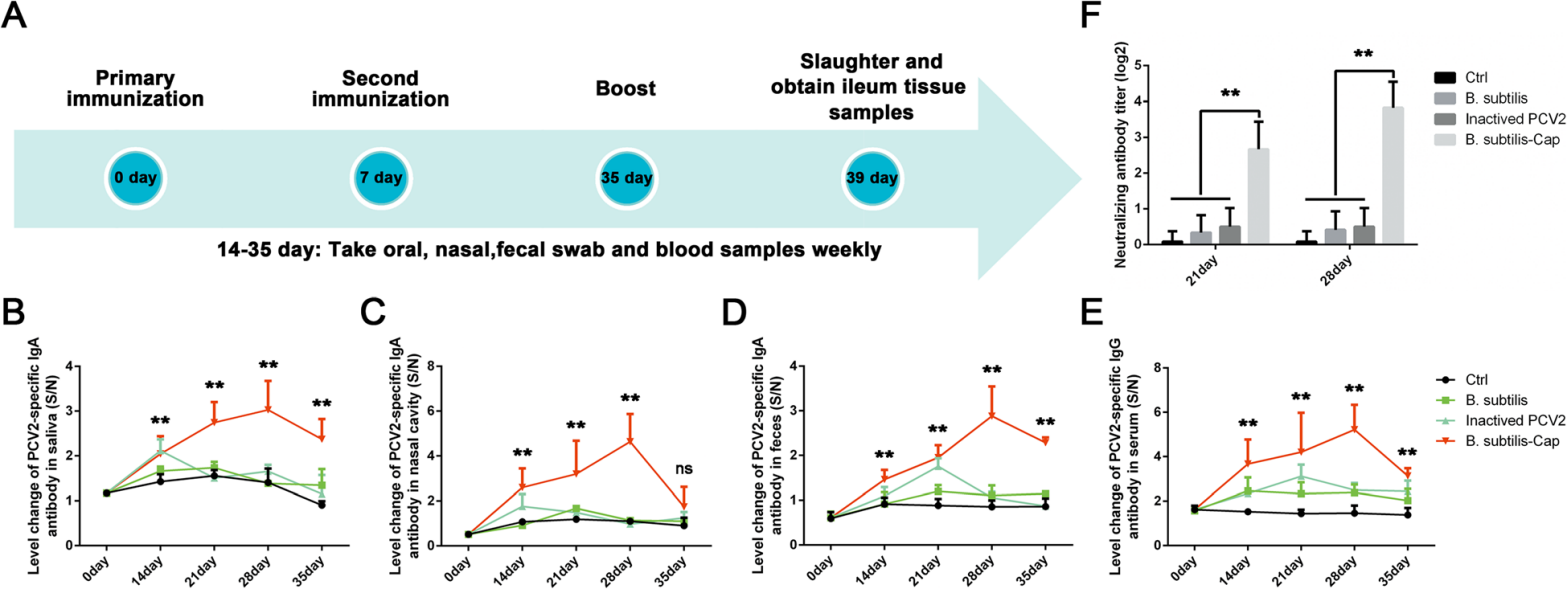
Immune response in piglets orally inoculated with *B. subtilis*-Cap. **a** Flow chart of inoculation and sample collection time course. Piglets were orally inoculated with *B. subtilis*-Cap, *B. subtilis*, inactivated PCV2, and PBS on day 0 and 7. Saliva, nasal swab, feces, and serum were collected as indicated and analyzed by ELISA for PCV2-specific IgA and IgG. **b** Levels of PCV2-specific IgA in the oral cavity. **c** Levels of PCV2-specific IgA in the intranasal cavity. **d** Levels of PCV2-specific IgA in feces. **e** Levels of PCV2-specific IgG in serum. **f** The results of neutralizing titer activities of swine serum antibody on day 21 and day 28, expressed as the reciprocal of the highest dilution of serum causing a 50% reduction in fluorescence. Data shown are the means ± S.D. of six samples. * 0.01 < *p* < 0.05, ** *p* < 0.01 (compared to the Ctrl group). The error bars represent standard deviations

We then repeated this experiment in piglets. As shown in Fig. 3, the levels of PCV2-specific IgA antibody in saliva, nasal cavities, and feces, of piglets inoculated with *B. subtilis*-Cap rose steadily over the time course of the experiment and were for the most part significantly increased over inactivated PCV2 throughout the experiment. In all but the nasal cavity samples, the levels of IgA in the piglets inoculated with *B. subtilis*-Cap remained elevated throughout the 35-day experiment, while in those inoculated with inactivated PCV2, IgA levels rose initially then dropped to background. PCV2-specific IgG levels in sera from *B. subtilis*-Cap inoculated piglets were significantly higher than from inactivated PCV2 inoculated piglets throughout the experiment. Furthermore, serum samples on day 21 and day 28 were further evaluated for the ability to neutralize PCV2 in vitro by serum neutralization assays. As shown in Fig. 3f, neutralizing antibody (NA) titers against PCV2 in the recombinant *B. subtilis*-Cap group were significantly higher than sera from other groups (*P* < 0.01). Swine immunized with recombinant *B. subtilis*-Cap developed mean NA titers of 1:6.3 on day 21 and 1: 14.3 on day 28. Sera from swine treated with PBS in Ctrl group was showed no neutralizing antibody activity, and sera from *B. subtilis* and Inactived PCV2 groups were showed a lower level of NA titers. These results demonstrate the effectiveness of *B. subtilis* as a viral vector for oral PCV2 vaccine delivery.

### Local cellular immune responses in ileums after oral immunization

**Fig. 4:**
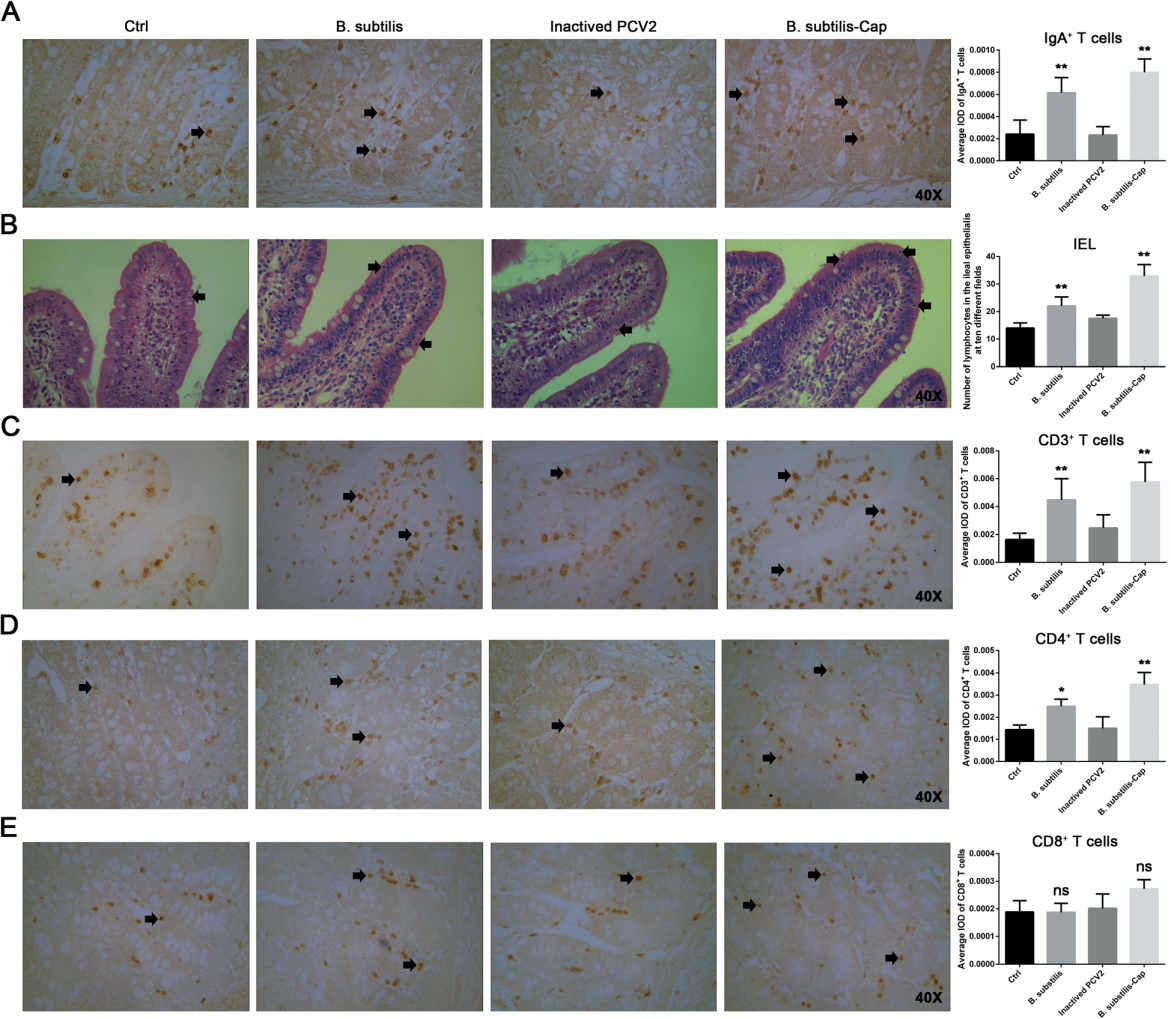
Effect of orally administered recombinant *B. subtilis*-Cap on ileal immunocompetent cells**.** Piglets were immunized as described above, then boosted on day 35 and sacrificed on the day 39. Ileum tissues were fixed with Bonn’s liquid, embedded in paraffin and sectioned to 5 μm thickness, the sections were subjected to hematoxylin–eosin staining or immunohistochemistry. **a** Distribution of IgA-secreting cells in piglet ileums (Magnification, × 40) and the average IOD of IgA-secreting cells distributed in piglet ileums. The IgA-secreting plasma cells, rounded with a deep yellow-brown nucleus (black arrows), were located mainly in the submucosa. **b** Distribution of IELs in ileums (Magnification, × 40) and the average of number of IELs counted per dose group. The IELs (black arrows) are located between intestinal epithelial cells, and beneath the intercellular tight junctions at the basal region of the epithelium. **c**, **d**, and **e** Distribution of CD3^+^, CD4^+^, and CD8^+^ T lymphocytes in piglet ileums (Magnification, × 40). The average IOD of CD3^+^, CD4^+^ and CD8^+^ T lymphocytes in the ileums are displayed in the histogram. Photomicrographs showing representative CD3^+^, CD4^+^ and CD8^+^ T lymphocytes which are stained a deep yellow-brown (black arrows). The average IOD was calculated from ten separate fields of ileum from each piglet, using Image-Pro Plus 6.0 software. The error bars represent standard deviations. * 0.01 < *p* < 0.05, ** *p* < 0.01 (compared to the Ctrl group)

Because the levels of mucosal PCV2-specific IgA antibody increased significantly following inoculation with recombinant *B. subtilis*-Cap, we next looked at the IgA-secreting cells in the piglets’ ileums by immunohistochemistry. We found that the average integrated optical density (IOD) of IgA-producing cells in ileums were increased significantly (*P* < 0.01) after stimulation with recombinant *B. subtilis*-Cap (Fig. 4a). The number of intestinal intraepithelial lymphocytes (IELs) in the ileums of *B. subtilis*-Cap inoculated piglets were significantly higher (*P* < 0.01, Fig. 4b) than in the control piglets. IELs, found in the epithelial layer of mucosal tissues, are involved in the recognition of and defense against pathogens. The average IOD of CD3^+^ and CD4^+^ T lymphocytes in the ileums of *B. subtilis*-Cap inoculated piglets were significantly higher than in the control piglets (*P* < 0.01, Fig. 4c and d). In contrast, CD8^+^ T lymphocytes did not appear to be stimulated in any of the experimental piglets (*P* > 0.05, Fig. 4e). These results demonstrated that oral immunization with recombinant *B. subtilis*-Cap resulted in increased lymphocytes and a robust immune response in the ileums of piglets.

### Levels of TLRs and cytokines

**Fig. 5:**
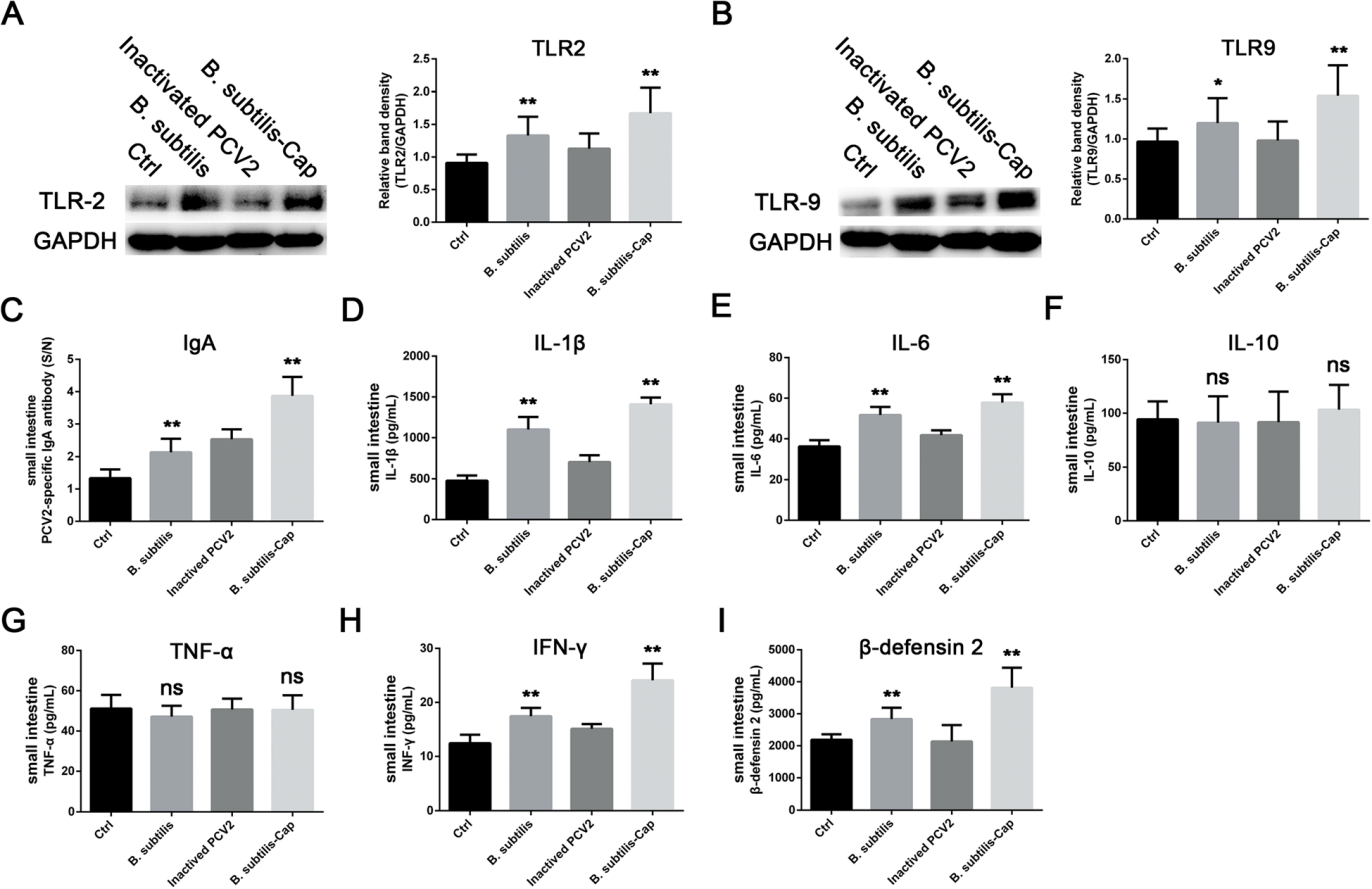
The expression levels of Toll-like receptors (TLRs), IgA, and cytokines**.** Associated immune factors were measured in ileum homogenates prepared on day 39 of the experiment (at slaughter). **a** TLR2 protein expression, the ratios of TLR2 to GAPDH were normalized to the control. **b** TLR9 protein expression, the ratios of TLR9 to GAPDH were normalized to the control. **c** IgA expression. **d-h** Cytokine IL1β, IL-6, IL-10, TNF-α, and IFN-γ expression. **i** Porcine β-defensin 2 expression. The error bars represent standard deviations. * 0.01 < *p* < 0.05, ** *p* < 0.01 (compared to the Ctrl group)

To further investigate the mucosal-cell immune response after oral inoculation, we tested for expression of TLR2, TLR9, IgA and associated cytokines in the ileum homogenates prepared after piglets were sacrificed. In piglets dosed with *B. subtilis*-Cap, levels of TLR2 (Fig. 5a) and TLR9 (Fig. 5b) protein, as well as IgA (Fig. 5c) were increased significantly over controls (*P* < 0.01). Activation of the immune response is usually associated with the secretion of cytokines; compared with piglets immunized with PBS or inactivated PCV2, the ileum homogenates from piglets immunized with *B. subtilis* and *B. subtilis*-Cap had significantly (*P* < 0.01) increased levels of IL-1β (Fig. 5d), IL-6 (Fig. 5e), IFN-γ (Fig. 5h), and β-defensin 2 (Fig. 5i), which is crucial for the innate immune system of gut protection owing to its antimicrobial, antiviral, and immunomodulatory activities [52, 53]. Levels of IL-10 (Fig. 5f) and TNF-α (Fig. 5g) were unchanged (*P* > 0.05).

## Discussion

Porcine circovirus type 2 (PCV2), the etiologic agent of post-weaning multi-systemic wasting syndrome (PMWS) has become a considerable economic burden to swine industries worldwide. PCV2 infects animals through the mucosal tissues of their respiratory and intestinal tracts [21–24]. Current vaccines administered parenterally induce neutralizing IgG antibodies in the serum, which may not play a substantial role in protecting against PCV2 infection since protection is primarily dependent on secretory IgA antibodies. The control of PCV2 infection requires a mucosal vaccine with a safe and effective agent; to address this need we constructed a recombinant *B. subtilis* that expresses the capsid protein of PCV2, *B. subtilis*-Cap. The capsid protein is the most immunogenic of PCV2 [6–8], and *B. subtilis* is widely used as a live vaccine vehicle for heterologous antigen delivery. It effectively elicits mucosal and systemic immune responses [42, 54–56], and itself possesses adjuvant-like properties enhancing the level of local and systemic immune responses [37, 57, 58]. Wang et al. reported that in mice, oral vaccination with *Lactococcus lactis* expressing PCV2 capsid protein resulted in enhanced expression of PCV2-specific IgA, there was however no effect on IgG expression [59]. In our study, piglets orally vaccinated with *B. subtilis*-Cap had induced robust humoral immune response by increasing levels of PCV2-specific IgA and IgG compared with piglets orally vaccinated with inactivated PCV2.

Dendritic cells are antigen-presenting cells widely distributed in the intestinal submucosa, they play a crucial role in linking innate and adaptive immunities [60, 61]. The development of DCs include immature stage (iDCs) and mature stage (mDCs). IDCs are specialized in antigen capture and processing, and are characterized by low expression of MHCII and co-stimulatory molecules on their surface. Upon contacting an antigen they activate into mature DCs and up-regulate antigen presentation associated factors and ultimately stimulate specific T cell immune responses [48, 62, 63]. Previous studies have demonstrated that *B. subtilis* enhances the expression of chemokine CCL20 in intestinal epithelial cells, recruits immature DCs into the intestinal epithelium, stimulates mature DC migration to mesenteric lymph nodes [64, 65]. It has also been reported that PCV2-induced immunosuppressive effects are owing to the specific silencing of plasmacytoid DCs, resulting in their failure to deliver pathogen-associated ‘danger’ signals [66]. Here we found that *B. subtilis*-Cap enhanced the MHC II and co-stimulatory molecules of BM-DCs. In addition, DCs sensitized by *B. subtilis*-Cap stimulated the proliferation of T lymphocytes. These results demonstrate that exposure to *B. subtilis*-Cap caused the maturation of DCs and the induction of the attendant immune response.

Secretory IgA (sIgA) is a major contributor to host defense at mucosal surfaces and plays a critical role in mucosal immunity. sIgA maintains mucosal homeostasis by regulating the composition of intestinal microflora and limiting the acute local inflammation induced by the invasion and colonization of pathogens [67, 68]. Serum IgG is the most abundant immunoglobulin in the blood, is vital for systemic immunity. Our experiments showed that oral immunization with *B. subtilis*-Cap resulted in elevated PCV2-specific IgA antibodies in saliva, nasal cavity, ileum and feces, as well as elevated PCV2-specific IgG in serum, demonstrating that oral immunization can elicit both mucosal and systemic immune responses effectively.

*B. subtilis* itself not only broadens the antibody response, but also increases T cell responses [36]. Intestinal intraepithelial lymphocytes are sentinel T lymphocytes in the intestinal tract, functioning in immune surveillance and defense against pathogens [69–71]. CD3^+^ T lymphocytes are mainly distributed in the lamina propria of the ileum, while CD4^+^ T cells (helper T cells) enhance the cytotoxicity mediated by CD8^+^ T cells. The ratio of CD4^+^ T cells to CD8^+^ T cells are an important factor in determining immunity states and levels [72, 73], and plays a central role in the induction of efficient immune responses against different diseases such as human immunodeficiency virus (HIV) and cancer [11–14]. Consist to previous study [74], the ratio of CD4^+^ T cells to CD8^+^ T cells was also increased significantly in mice immunized with *B. subtilis-*Cap. We found that in the intestinal tract of piglets orally administration of *B. subtilis-*Cap, the number of IgA producing plasma cells, IELs, CD3^+^, and CD4^+^ T lymphocytes were significantly increased. Toll-like receptors (TLR) activation also plays an important role in regulating mucosal immune responses in the intestine. TLRs are major class of pattern recognition receptors that recognize specific molecules generally shared by pathogens including virus, bacteria, and fungi [75–79]. *B. subtilis* is recognized by TLR2 and TLR9 which triggers both innate and adaptive immune responses, eliciting the production of immunomodulatory cytokines and chemokines, and upregulating the co-stimulatory molecules of DCs [37, 40, 58, 80, 81]. We found that in the ileal cells of piglets orally administered *B. subtilis*-Cap, expression of TLR2 and TLR9 was significantly elevated, as was secretion of IL-1β, IL-6, and IFN-γ. Among the experimental piglets, there were however no significant differences in the expression of IL-10 or TNF-α, which may due to inhibition by IL-6. *B. subtilis*-Cap immunization also resulted in increased β-defensin-2 levels, which is beneficial for the growth performance and improve the intestinal health.

## Conclusions

Recombinant *B. subtilis*-Cap stimulated the maturation of BM-DCs in vitro. In piglets orally immunized with *B. subtilis*-Cap, humoral and cellular immunity was enhanced as demonstrated by the upregulation of PCV2-specific IgA and IgG antibodies. These results indicate that recombinant *B. subtilis*-Cap is a promising candidate in the effort to develop new PCV2 vaccines.

## Supplementary information

**Additional file 1: Figure S1.** Effect on CD4^+^ to CD8^+^ T cells. Mesenteric lymph node cells isolated from healthy mice 35 days after inoculation were cultured in lymphocyte culture medium at 2 × 10^5^ cells per well in 24-well culture plates, and stimulated by recombinant *B. subtilis*-Cap for 72 h. Non-stimulated cells were used as negative controls. **a** and **b** The percentage of CD3^+^ T cells (Q2). **c** The gates were based on CD3^+^ T cell results (Q2), then further gated for CD4^+^ and CD8^+^ T cells. **d** The ratio of CD4^+^ to CD8^+^ T cells for each group. The error bars represent standard deviations. * 0.01 < *p* < 0.05, ** *p* < 0.01 (compared to the Ctrl group).

**Additional file 2: Figure S2.** Mice were inoculated orally with *B. subtilis*, *B. subtilis*-Cap, inactivated PCV2, and PBS on days 0 and 7. Intestinal fluids and sera were collected on days 14, 21, 28, and 35. Endpoint titers (or absorbance at 450 nm) of PCV2-specific intestinal IgA and serum IgG antibodies were investigated by ELISA. **a** Levels of PCV2-specific IgA in intestinal fluids. **b** Levels of PCV2-specific IgG antibody in serum. The error bars represent standard deviations. * 0.01 < *p* < 0.05, ** *p* < 0.01 (compared to the Ctrl group).

## Data Availability

All data generated or analyzed during this study are included either in this article or in the supplementary Materials and Methods, Tables, Figures and Figure Legends files.

## Abbreviations

*B. subtilis*: *Bacillus subtilis*
BM-DCs: Bone marrow-derived dendritic cells
Cap: Capsid protein
PCV2: Porcine circovirus type 2
