# Supplementary material for: Immune response in piglets orally immunized with recombinant Bacillus subtilis expressing the capsid protein of porcine circovirus type 2
Source: Cell Commun Signal. 2020 Feb 11;18:23. doi: 10.1186/s12964-020-0514-4 (PMC7014726; doi:10.1186/s12964-020-0514-4)
Supplement: Supplementary file 1 — Additional file 1: Figure S1. Effect on CD4+ to CD8+ T cells. Mesenteric lymph node cells isolated from healthy mice 35 days after inoculation were cultured in lymphocyte culture medium at 2 × 10^5^ cells per well in 24-well culture plates, and stimulated by recombinant B. subtilis-Cap for 72 h. Non-stimulated cells were used as negative controls. a and b The percentage of CD3+ T cells (Q2). c The gates were based on CD3+ T cell results (Q2), then further gated for CD4+ and CD8+ T cells. d The ratio of CD4+ to CD8+ T cells for each group. The error bars represent standard deviations. * 0.01 < p < 0.05, ** p < 0.01 (compared to the Ctrl group). [file 12964_2020_514_MOESM1_ESM.docx]

[Additional file 1:](https://static-content.springer.com/esm/art%3A10.1186%2Fs12964-019-0429-0/MediaObjects/12964_2019_429_MOESM1_ESM.docx)

**
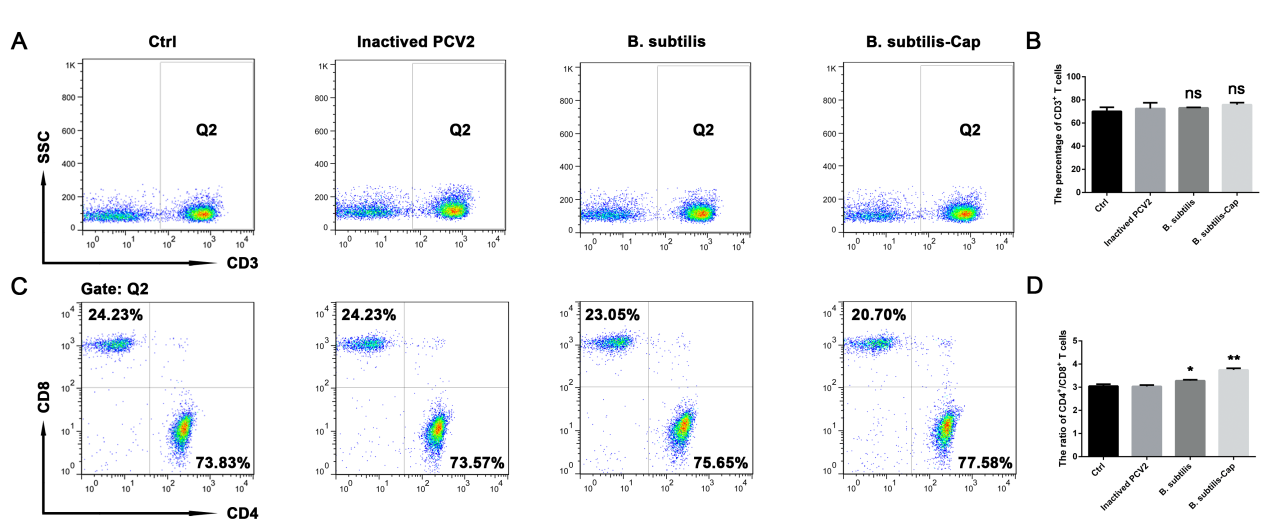
**

**Figure S1.** Effect on CD4^+^ to CD8^+^ T cells. Mesenteric lymph node cells isolated from healthy mice 35 days after inoculation were cultured in lymphocyte culture medium at 2×10^5^ cells per well in 24-well culture plates, and stimulated by recombinant *B. subtilis*-Cap for 72 h. Non-stimulated cells were used as negative controls. **a** and **b** The percentage of CD3^+^ T cells (Q2). **c** The gates were based on CD3^+^ T cell results (Q2), then further gated for CD4^+^ and CD8^+^ T cells. **d** The ratio of CD4^+^ to CD8^+^ T cells for each group. The error bars represent standard deviations. * 0.01 < p < 0.05, ** p < 0.01 (compared to the Ctrl group).
