# Supplementary material for: Immune response in piglets orally immunized with recombinant Bacillus subtilis expressing the capsid protein of porcine circovirus type 2
Source: Cell Commun Signal. 2020 Feb 11;18:23. doi: 10.1186/s12964-020-0514-4 (PMC7014726; doi:10.1186/s12964-020-0514-4)
Supplement: Supplementary file 2 — Additional file 2: Figure S2. Mice were inoculated orally with B. subtilis, B. subtilis-Cap, inactivated PCV2, and PBS on days 0 and 7. Intestinal fluids and sera were collected on days 14, 21, 28, and 35. Endpoint titers (or absorbance at 450 nm) of PCV2-specific intestinal IgA and serum IgG antibodies were investigated by ELISA. a Levels of PCV2-specific IgA in intestinal fluids. b Levels of PCV2-specific IgG antibody in serum. The error bars represent standard deviations. * 0.01 < p < 0.05, ** p < 0.01 (compared to the Ctrl group). [file 12964_2020_514_MOESM2_ESM.docx]

[Additional file 2:](https://static-content.springer.com/esm/art%3A10.1186%2Fs12964-019-0429-0/MediaObjects/12964_2019_429_MOESM1_ESM.docx)

**
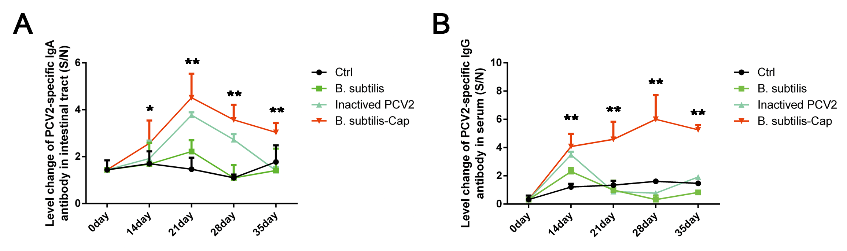
**

**Figure S2.** Mice were inoculated orally with *B. subtilis*, *B. subtilis*-Cap, inactivated PCV2, and PBS on days 0 and 7. Intestinal fluids and sera were collected on days 14, 21, 28, and 35. Endpoint titers (or absorbance at 450 nm) of PCV2-specific intestinal IgA and serum IgG antibodies were investigated by ELISA. **a** Levels of PCV2-specific IgA in intestinal fluids. **b** Levels of PCV2-specific IgG antibody in serum. The error bars represent standard deviations. * 0.01 < p < 0.05, ** p < 0.01 (compared to the Ctrl group).
